# Supplementary material for: Seal milk oligosaccharides rival human milk complexity and exhibit functional dynamics during lactation
Source: Nat Commun. 2025 Nov 25;16:10067. doi: 10.1038/s41467-025-66075-2 (PMC12647773; doi:10.1038/s41467-025-66075-2)
Supplement: Supplementary file 2 — Description of Additional Supplementary Files [file 41467_2025_66075_MOESM2_ESM.pdf]

Description of Additional Supplementary Files

Supplementary Data 1: The full milk glycome of Halichoerus grypus. For all structurally characterized glycans from the milk of grey seals, we denote their glycan sequence, as determined by LC-MS/MS and the rest of our workflow, as well as the information whether they represent novel structures, when compared to a pan-mammalian database.

Supplementary Data 2: New motifs in seal milk oligosaccharides impact lectin binding. For three novel structural epitopes (sulfated 2'-fucosyllactose and proximal as well as distal type-2 sialyl H-antigen, we aggregated glycan array binding information from glycowork, for the minimal glycans carrying these epitopes and a closely related control structure. Binding to lectins is shown in median z-scores of relative fluorescence units and only binding to lectins is shown where both structures have been measured.

Supplementary Data 3: The largest so far characterized milk oligosaccharides reside in grey seal milk. We show here all glycans from Supplementary Table 1 that contain more than 18 monosaccharides (the currently largest characterized milk oligosaccharide). These newly discovered structures reach up to 28 monosaccharides.

Supplementary Data 4: Largest characterized mammalian glycans overall. All fully characterized, currently known glycans comprising more than 20 monosaccharides and occurring in mammals are shown, drawn from the df_glycan database within glycowork (v1.5).

Supplementary Data 5: LacdiNAc-containing glycans in the milk of Halichoerus grypus. Here, we provide the sequences of all characterized, LacdiNAc-carrying milk oligosaccharides from grey seals, as well as the information whether they represent novel structures, when compared to a pan-mammalian database.

Supplementary Data 6: Sulfated glycans in the milk of Halichoerus grypus. Here, we provide the sequences of all characterized, sulfated milk oligosaccharides from grey seals, as well as the information whether they represent novel structures, when compared to a pan-mammalian database.

Supplementary Data 7: Quantification of structurally characterized milk oligosaccharides during seal lactation. For five seals (SA to SE) and four timepoints (d2, d7, d13, and d17/18/19), we here provide the relative abundances of all quantified milk oligosaccharides, along with their sequence via IUPAC-condensed.

Supplementary Data 8: Transformed abundances of milk oligosaccharides during seal lactation. For five seals (SA to SE) and four timepoints (d2, d7, d13, and d17/18/19), we here provide the center log ratio (CLR)-transformed relative abundances of all quantified milk oligosaccharides, along with their sequence via IUPAC-condensed.

Supplementary Data 9: The changing milk metabolome of grey seals. For five seals (SA to SE) and four timepoints (d2, d7, d13, and d17/18/19), we here provide the relative ion intensity of all quantified milk metabolites from Watson et al., 2021, along with their descriptor.

Supplementary Data 10: Correlations across milk oligosaccharide features and metabolites. Shown are the significant regularized partial correlations between milk oligosaccharide substructures (featurized via the "known" and "exhaustive" keywords in glycowork) and metabolites. All correlations were calculated from CLR-transformed glycomics and metabolomics data.

Supplementary Data 11: Cytokine assay of milk oligosaccharide effect on macrophage activity.

Supplementary Data 12: LC-ESI-MS/MS detection of neutral milk oligosaccharides in deposited raw files. All detected compositions are provided with their retention times and intensities across all files.

Supplementary Data 13: LC-ESI-MS/MS detection of acidic milk oligosaccharides in deposited raw files. All detected compositions are provided with their retention times and intensities across all files.
