# Supplementary material for: Seal milk oligosaccharides rival human milk complexity and exhibit functional dynamics during lactation
Source: Nat Commun. 2025 Nov 25;16:10067. doi: 10.1038/s41467-025-66075-2 (PMC12647773; doi:10.1038/s41467-025-66075-2)
Supplement: Supplementary file 4 — Reporting Summary [file 41467_2025_66075_MOESM4_ESM.pdf]

nature portfolio

Corresponding author(s): Daniel Bojar

Last updated by author(s): Sep 17, 2025

Reporting Summary

Nature Portfolio wishes to improve the reproducibility of the work that we publish. This form provides structure for consistency and transparency in reporting. For further information on Nature Portfolio policies, see our Editorial Policies and the Editorial Policy Checklist.

Statistics

For all statistical analyses, confirm that the following items are present in the figure legend, table legend, main text, or Methods section.

n/a	Confirmed	
☐	☒	The exact sample size (*n*) for each experimental group/condition, given as a discrete number and unit of measurement
☐	☒	A statement on whether measurements were taken from distinct samples or whether the same sample was measured repeatedly
☐	☒	The statistical test(s) used AND whether they are one- or two-sided<br>*Only common tests should be described solely by name; describe more complex techniques in the Methods section.*
☐	☒	A description of all covariates tested
☐	☒	A description of any assumptions or corrections, such as tests of normality and adjustment for multiple comparisons
☐	☒	A full description of the statistical parameters including central tendency (e.g. means) or other basic estimates (e.g. regression coefficient) AND variation (e.g. standard deviation) or associated estimates of uncertainty (e.g. confidence intervals)
☐	☒	For null hypothesis testing, the test statistic (e.g. *F*, *t*, *r*) with confidence intervals, effect sizes, degrees of freedom and *P* value noted<br>*Give P values as exact values whenever suitable.*
☒	☐	For Bayesian analysis, information on the choice of priors and Markov chain Monte Carlo settings
☒	☐	For hierarchical and complex designs, identification of the appropriate level for tests and full reporting of outcomes
☐	☒	Estimates of effect sizes (e.g. Cohen's *d*, Pearson's *r*), indicating how they were calculated

Our web collection on statistics for biologists contains articles on many of the points above.

Software and code

Policy information about availability of computer code

Data collection	Glycomics data acquisition and processing employed Xcalibur software (Version 2.0.7), followed by quantification via Progenesis QI (Nonlinear Dynamics Ltd).
Data analysis	All glycomics data analysis was performed using glycowork (v1.5; https://github.com/BojarLab/glycowork), the statsmodels package (version 0.14) and the scipy package (version 1.11) in Python 3.12.6. Cytokine multiplex experiments were analyzed with the LEGENDplex Data Analysis Software Suite (Biolegend, Qognit).

For manuscripts utilizing custom algorithms or software that are central to the research but not yet described in published literature, software must be made available to editors and reviewers. We strongly encourage code deposition in a community repository (e.g. GitHub). See the Nature Portfolio guidelines for submitting code & software for further information.

Data

Policy information about availability of data

All manuscripts must include a data availability statement. This statement should provide the following information, where applicable:

- Accession codes, unique identifiers, or web links for publicly available datasets
- A description of any restrictions on data availability
- For clinical datasets or third party data, please ensure that the statement adheres to our policy

Unless otherwise stated, all data supporting the results of this study can be found in the article, supplementary, and source data files. All processed data generated and/or used in this article can either be found in supplemental tables or as stored datasets within glycowork (23). The glycomics MS raw files have been deposited in

the GlycoPOST database under the ID GPST000556 [https://glycopost.glycosmos.org/entry/GPST000556], with the corresponding retention times and compositions found in Tables S12-13. Source Data are provided with this paper.

Research involving human participants, their data, or biological material

Policy information about studies with human participants or human data. See also policy information about sex, gender (identity/presentation), and sexual orientation and race, ethnicity and racism.

Reporting on sex and gender	-
Reporting on race, ethnicity, or other socially relevant groupings	-
Population characteristics	-
Recruitment	-
Ethics oversight	-

Note that full information on the approval of the study protocol must also be provided in the manuscript.

Field-specific reporting

Please select the one below that is the best fit for your research. If you are not sure, read the appropriate sections before making your selection.

- [x] Life sciences
- [ ] Behavioural & social sciences
- [ ] Ecological, evolutionary & environmental sciences

For a reference copy of the document with all sections, see nature.com/documents/nr-reporting-summary-flat.pdf

Life sciences study design

All studies must disclose on these points even when the disclosure is negative.

Sample size	No prior sample-size calculation was performed as we obtained those samples from other researchers who have collected them for a previous study. Working with wild mammals and relying on them producing offspring that season has limited the number of collected samples, yet the resulting sample size represents a common and sufficient size of typical glycomics datasets. Only female seals were considered for sampling, since they are the only lactating sex in grey seals. For functional milk oligosaccharide experiments, we oriented ourselves at our prior work (doi: 10.1016/j.mcpro.2023.100635), in which a sample size of three biological replicates was sufficient to observe differences between conditions.
Data exclusions	No data were excluded
Replication	All functional experiments of milk oligosaccharides in our study were reproducible (both with three technical and three biological replicates)
Randomization	Not relevant to our study, as we do not compare different experimental groups
Blinding	Not relevant to our study, as we do not analyze different treatments etc.

Reporting for specific materials, systems and methods

We require information from authors about some types of materials, experimental systems and methods used in many studies. Here, indicate whether each material, system or method listed is relevant to your study. If you are not sure if a list item applies to your research, read the appropriate section before selecting a response.

Materials & experimental systems

n/a	Involved in the study	
x		Antibodies
	x	Eukaryotic cell lines
x		Palaeontology and archaeology
	x	Animals and other organisms
x		Clinical data
x		Dual use research of concern
x		Plants

Methods

n/a	Involved in the study	
x		ChIP-seq
x		Flow cytometry
x		MRI-based neuroimaging

Eukaryotic cell lines

Policy information about cell lines and Sex and Gender in Research

Cell line source(s)	THP-1 (CVCL_0006) is a male human monocyte cell line isolated from peripheral blood of an acute monocytic leukemia patient. We obtained this cell line from ATCC (cat. no. TIB-202)
Authentication	All experiments were performed on freshly thawed cells after equilibration and differentiation as described in the manuscript. A successful differentiation reaction indicated the status of the provided cells as monocytes. The cells were not otherwise authenticated.
Mycoplasma contamination	All cell lines tested negative for mycoplasma contamination
Commonly misidentified lines (See ICLAC register)	-

Animals and other research organisms

Policy information about studies involving animals; ARRIVE guidelines recommended for reporting animal research, and Sex and Gender in Research

Laboratory animals	The study did not involve laboratory animals
Wild animals	Female subjects of Halichoerus grypus (grey seal) received weight-proportioned anesthesia using Zoletil 100® (Virbac, Bury St Edmunds, Suffolk, UK), after which intravenous oxytocin was administered to induce milk ejection, followed by intramuscular tetracycline as a preventive antibiotic measure. The oxytocin treatment consisted of a 1 mL intravenous dose (10 iu mL^{-1} or 0.18 mg mL^{-1}; Oxytocin-S, Intervet UK). Given that post-parturition female grey seals in this population typically weighed approximately 180 kg, the resulting oxytocin dosage was calculated at roughly 1 µg kg^{-1}.
Reporting on sex	Only female seals were considered for sampling, since they are the only lactating sex in grey seals.
Field-collected samples	Milk samples were originally collected in a previous study (17) from a seal colony on the Isle of May, Scotland.
Ethics oversight	Work involving animals in this study complied with all relevant ethical regulations and was licensed under the UK Home Office project 60/4009 or preceding versions and conformed to the UK Animals (Scientific Procedures) Act, 1986. Research was approved by the University of St Andrews Animal Welfare and Ethics Committee.

Note that full information on the approval of the study protocol must also be provided in the manuscript.

Plants

Seed stocks	-
Novel plant genotypes	-
Authentication	-
